# Assessment of micronecrotic tumor tissue using dynamic contrast-enhanced magnetic resonance imaging

**DOI:** 10.1016/j.ejmp.2017.01.010

**Published:** 2017-02

**Authors:** Olga Schimpf, Stefan Hindel, Lutz Lüdemann

**Affiliations:** Department of Radiation Therapy, Hufelandstr. 55, Universitätsklinikum Essen, 45147 Essen, Germany

**Keywords:** DCE-MRI, Diffusion, Contrast agent, Interstitium, Tumor, Necrotic tissue

## Abstract

Compartmental models for evaluation of dynamic contrast-enhanced magnetic resonance imaging (DCE-MRI) datasets assume a homogeneous interstitital volume distribution and homogeneous contrast agent (CA) distribution within each compartment, neglecting effects of CA diffusion within the compartments. When necrotic or micronecrotic tumor tissue is present, these assumptions may no longer be valid. Therefore, the present study investigates the validity of three compartmental models in assessing tumors with necrotic components.

The general diffusion equation for inhomogeneous tissue was used to simulate the extravasation of a low-molecular-weight contrast agent from a feeding vessel into the interstitial space. The simulated concentration-time curves were evaluated using the extended Tofts model, a parallel 3-compartment model, and a sequential 3-compartment model.

The extended Tofts model overestimated the interstitial volume fraction by a median of 6.9% resp. 10.0% and the parallel 3-compartment model by 8.6% resp. 15.5%, while the sequential 3-compartment model overestimated it by 0.2% resp. underestimated it by 18.8% when simulating a mean vessel distance of 100 μm resp. 150 μm. Overall, the sequential 3-compartment model provided more reliable results both for the total fractional interstitial volume and for the interstitial subcompartments.

## Highlights

• Contrast agent diffusion in inhomogeneous tissue using a macroscopic model is described.
• Methodology of diffusion simulation in an inhomogeneous tissue is presented.
• The impact of necrotic tumor tissue on contrast enhancement is investigated.
• Why compartment models may provide an interstitial volume >100.
• The possibility to assess necrotic tumor tissue using DCE-MRI is demonstrated.

## 1 Introduction

Dynamic contrast-enhanced magnetic resonance imaging (DCE-MRI) typically uses low-molecular-weight contrast agents (LMWCAs). These LMWCAs extravasate instantaniously into the interstitial space, also known as the extravascular extracellular space. To assess enhancement after contrast agent administration, several pharmacokinetic compartmental models are used [1], [2]. The most popular pharmacokinetic compartmental model is the so-called Tofts model [3], which uses a transfer constant to assess contrast enhancement. All of these models assume a homogeneous tracer concentration within each compartment and do not take diffusion effects into account. Thus, the permeability surface product of the supporting vascular network obtained with use of compartmental model is far from reflecting the true value [4].

Another parameter assessed by DCE-MRI is the fractional interstitial volume. This is an important and widely used parameter because changes in the interstitial volume of a tissue can indicate pathology. Tumors, in particular, have a markedly altered interstitial volume compared with healthy tissue, and the interstitial volume can vary within a tumor. Angiogenesis in a tumor is irregular, resulting in inhomogeneous oxygen supply across the tumor with development of micronecrotic and hypoxic tissue in areas receiving less oxygen [5], [6]. Typically, tissue oxygen supply decreases with the distance from blood vessels. Therefore, tissue near vessels receives enough oxygen, while tissue farther away becomes hypoxic and tissue even farther away undergoes necrosis [7], [8]. Such a tumor thus varies in relative interstitial tissue volumes with necrotic components having a markedly higher interstitial volume and a slightly higher diffusion coefficient.

Hypoxic areas in a tumor lower its sensitivity to radiation, thus considerably limiting the benefit of radiotherapy [9], [10], [11], [12]. This has been discussed as a possible reason for the still rather poor local control rate of advanced head and neck tumors even in patients treated with an optimal combination of radiotherapy and chemotherapy [13] [14]. For prostate cancer, it has been shown that dose escalation in hypoxic areas is beneficial [15].

While pharmacokinetic compartmental models fail to provide reliable values for the permeability surface product, they perform much better in quantifying the fractional interstitial volume. Tissue with late contrast enhancement is potentially necrotic or micronecrotic because it is so far away from the nearest vessel that the diffusion distance of oxygen is exceeded [16]. As a result, it takes very long for low-molecular-weight contrast agents to fill the interstitial space of necrotic tissue areas [17]. DCE-MRI thus allows identification of micronecrotic areas with increased interstitial volume [17] and long diffusion distances. Often compartment models provide realistic interstitial volume values, but sometimes authors calculated interstitial volumes larger than 100% [18]. Moreover, compartmental models sometimes also fail to provide true fractional interstitial volumes. This is especially a problem in case of slow uptake [18] by poorly perfused tissues. None of the currently available compartmental models takes an inhomogeneous interstitial volume into account. Therefore, their ability to assess the interstitial volume fraction in tumors with inhomogeneously perfused tissue remains unknown.

The aim of the present study is to assess how reliably different compartmental models determine interstitial volume fraction in the presence of an inhomogeneous interstitial volume distribution. Therefore, extravasation and distribution by diffusion of an LMWCA in the interstitial space were simulated. A simplified tissue model was designed consisting of a central area with a small interstitial volume and a peripheral area with a higher interstitial volume. Since the simulation parameters, e.g. fractional interstitial volume, were to be varied continuously, macroscopic tissue description was chosen [19]. Three models were evaluated, the extended Tofts model, a parallel 3-compartment model, and a sequential 3-compartment model. When a 3-compartment model is used, each tissue type is represented by one compartment.

## 2 Theory

### 2.1 Diffusion equation

Diffusion in the extracellular space of a homogeneous biological tissue can be excellently described by solving the macroscopic differential equation [4], [19], [20], [21]. For a porous medium, this is done by making several assumptions, namely that diffusion coefficient *D* and porosity ε are the same throughout the medium, while a tortuosity parameter, λ, is used to account for effects of porosity on diffusion processes. The indicator concentration in the interstitial volume, 〈*C*〉*_e_*, and thus the averaged diffusion equation for homogeneous porous media is given by the following equation [19]:

(1) $$\varepsilon \frac{\partial \langle C\rangle_{e}}{\partial t}=\frac{\varepsilon D}{\lambda^{2}}\nabla^{2}\langle C\rangle_{e}$$

However, since biological tissues are often inhomogeneous, we need a diffusion equation that also applies to inhomogeneous tissues, which are characterized by the fact that parameters such as the diffusion coefficient or relative interstitial volume vary with the spatial position within the tissue. The general diffusion equation for porous media has the following form [22]:

(2) $$\varepsilon \frac{\partial \langle C\rangle_{e}}{\partial t}=\nabla \cdot \left[\frac{\varepsilon D}{\lambda^{2}}\nabla \langle C\rangle_{e}\right]$$

Tortuosity λ accounts for restriction and deceleration of diffusion processes in biological tissues compared with free media. Tortuosity λ can be interpreted as a composite parameter accounting for both the longer geometric diffusion pathway around cells in the interstitial space and the effects of interstitial viscosity [23]:

(3) $$\lambda =\lambda_{g}\lambda_{v}\text{.}$$

$\lambda_{g}$ represents the geometric component of tortuosity and $\lambda_{v}$ its viscous component. Viscosity slows down diffusion through the presence of macromolecules in the interstitial space, which can become obstacles for diffusing particles [21]. The geometric effect of longer diffusion pathways in the extracellular space results from the tortuosity of diffusion pathways around cells [24]. Low-molecular-weight indicators do not enter cells, instead taking the longer routes around them. Geometric tortuosity is defined as the ratio of the actual effective pathway, $L_{\mathrm{eff}}$, between two points to the shortest distance, *L*, between the two points:

(4) $$\lambda_{g}=\frac{L_{\mathrm{eff}}}{L}$$

In an experimental setup for determining λ in a tissue of interest, one has to take both geometric effects and interstitial viscosity into account. This is accomplished by interpreting tortuosity in relation to diffusion coefficients *D* and $D_{\mathrm{eff}}$ [21]:

(5) $$\lambda =\sqrt{\frac{D}{D_{\mathrm{eff}}}}$$

Therefore, λ is determined by first measuring the diffusion coefficient, *D*, for a given molecule in free aqueous medium or gel and then comparing this value with the effective diffusion coefficient, $D_{\mathrm{eff}}$, measured for the same molecule in the target tissue. If diffusion is slowed or restricted, for instance due to the presence of cells in biological tissue that cannot be penetrated by a contrast agent, then λ>1. Other factors that can affect diffusion in the extracellular space include entrapment in so-called dead spaces, binding, and uptake of diffusing particles into the tissue [24], which are not taken into account here.

### 2.2 Relationship between tortuosity and porosity

The relationship between the parameters of tortuosity, λ, and porosity, ε, is important for describing diffusion in a porous medium. Several studies, primarily in the brain, have been conducted to elucidate the relationship between these two parameters [23], [25], [26]. Tao and Nicholson [25], for example, performed Monte Carlo simulation of 3D diffusion. They constructed three different extracellular space geometries to investigate effects of tissue geometry and structural properties on tortuosity, λ. For each of the three models, they performed simulations with different extracellular space volumes, ε. They found tortuosity λ to be independent of extracellular space geometry and, for all three models investigated, can be described by the following equation:

(6) $$\lambda =\sqrt{\frac{3-\varepsilon }{2}}$$

Mota et al. [23] analyzed published experimental diffusion data obtained in central nervous system tissue to derive a general relationship between λ and ε:

(7) $$\lambda =\varepsilon^{-n}\text{.}$$

In this equation, *n* is the index of tortuosity and depends on porosity ε of the medium investigated. Most values of index *n* for experimental diffusion data are distributed in the range Mota et al. [23] defined by the upper threshold of $n=0.23+0.3\varepsilon +\varepsilon^{2}$ and the lower threshold of $n=0.23+\varepsilon^{2}$ for *n*. In the present study, we use the mean value for *n*:

(8) $$n=0.23+0.15\varepsilon +\varepsilon^{2}\text{.}$$

Therefore, tortuosity can be calculated using the following equation, assuming that porosity is known:

(9) $$\lambda =\varepsilon^{-(0.23+0.15\varepsilon +\varepsilon^{2})}\text{.}$$

**Fig. 1:**
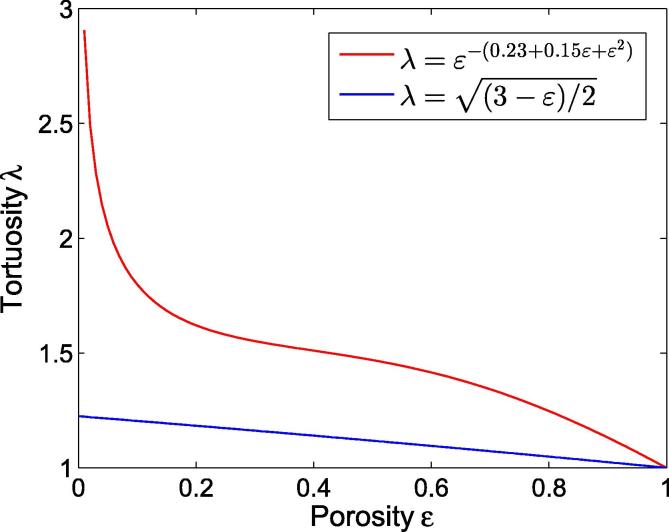
The relationship between tortuosity λ and porosity ε. The blue curve represents Eq. (6), which was derived from the simulation study of Tao and Nicholson [25], and only takes the geometric component of λ into account. The red curve represents Eq. (9), which was derived from results of experimental investigations of diffusion in brain tissue [23].

Fig. 1 represents Eqs. (6), (9) as curves.

It follows from Eq. (6) that λ increases with decreasing ε and can have a maximum value of λ=1.225 (see Fig. 1). However, experimental determination of λ in other studies [20], [24], [27] yielded a tortuosity λ of approx. 1.6 for the typical ε=0.2 in the brain. The deviation of λ determined with Eq. (6) from this value might be attributable to the fact that Tao and Nicholson only took geometric tissue effects into account, ignoring other factors, such as extracellular matrix or viscosity, which might also affect diffusion in the extracellular space. In contrast, the value of λ determined with Eq. (9) is in relatively good agreement with the typical value in the brain.

Since Mota et al. analyzed experimental data, we here use Eq. (9) for calculating λ. The relationship between porosity and tortuosity was primarily studied in brain tissue. However, it is assumed that the relationship between λ and ε is similar in other biological tissues.

### 2.3 Discretization of the diffusion equation

In this study, we exploit cylindrical symmetry around blood vessels to reduce diffusion calculation to the two-dimensional case. The diffusion equation for macroscopic concentration distribution for the two-dimensional case, for which we now use C(x,y,t), is as follows:

(10) $$\varepsilon \frac{\partial C}{\partial t}=\frac{\partial }{\partial x}\left[\varepsilon D_{\mathrm{eff}}\frac{\partial }{\partial x}C\right]+\frac{\partial }{\partial y}\left[\varepsilon D_{\mathrm{eff}}\frac{\partial }{\partial y}C\right]$$

Two-dimensional discretization of the diffusion equation is done applying discretization principles [28]. Supplementing time *t* discretization by grid width Δt, the space is additionally discretized in x- and y-direction by grid width Δy=Δx. Next, function C(x,y,t) is approximated by discrete function $C_{i\text{,}j}^{n}=C(x_{i}\text{,}y_{j}\text{,}t_{n})$; analogously, the variable coefficients are approximated by $A_{i\text{,}j}=A(x_{i}\text{,}y_{j})=\varepsilon (x_{i}\text{,}y_{j})D_{\mathrm{eff}}(x_{i}\text{,}y_{j})$. Using the forward difference quotient for time derivation and the central difference quotient for spatial derivations yields the following discretizations of Eq. (10):

(11) $$\varepsilon_{i\text{,}j}\frac{C_{i\text{,}j}^{n+1}-C_{i\text{,}j}^{n}}{\Delta t}=\frac{A_{i-\frac{1}{2}\text{,}j}C_{i-1\text{,}j}^{n}-(A_{i-\frac{1}{2}\text{,}j}+A_{i+\frac{1}{2}\text{,}j})C_{i\text{,}j}^{n}+A_{i+\frac{1}{2}\text{,}j}C_{i+1\text{,}j}^{n}}{\Delta x^{2}}+\frac{A_{i\text{,}j-\frac{1}{2}}C_{i\text{,}j-1}^{n}-(A_{i\text{,}j-\frac{1}{2}}+A_{i\text{,}j+\frac{1}{2}})C_{i\text{,}j}^{n}+A_{i\text{,}j+\frac{1}{2}}C_{i\text{,}j+1}^{n}}{\Delta y^{2}}$$

Next, several substitutions are made in Eq. (11): Δx=Δy,

$A_{i\pm \frac{1}{2}\text{,}j}=\frac{1}{2}\left(A_{i\text{,}j}+A_{i\pm 1\text{,}j}\right)$, and $A_{i\text{,}j\pm \frac{1}{2}}=\frac{1}{2}\left(A_{i\text{,}j\pm 1}+A_{i\text{,}j}\right)$. Substitution and transformation of Eq. (11) yields the difference scheme in two dimensions:

(12) $$\varepsilon_{i\text{,}j}C_{i\text{,}j}^{n+1}=\varepsilon_{i\text{,}j}C_{i\text{,}j}^{n}+\frac{\Delta t}{\Delta x^{2}}\left[\frac{1}{2}(A_{i-1\text{,}j}+A_{i\text{,}j})C_{i-1\text{,}j}^{n}+\frac{1}{2}(A_{i\text{,}j}+A_{i+1\text{,}j})C_{i+1\text{,}j}^{n}+\frac{1}{2}(A_{i\text{,}j-1}+A_{i\text{,}j})C_{i\text{,}j-1}^{n}+\frac{1}{2}(A_{i\text{,}j}+A_{i\text{,}j+1})C_{i\text{,}j+1}^{n}-\left(\frac{1}{2}(A_{i-1\text{,}j}+A_{i\text{,}j})+\frac{1}{2}(A_{i\text{,}j}+A_{i+1\text{,}j})+\frac{1}{2}(A_{i\text{,}j-1}+A_{i\text{,}j})+\frac{1}{2}(A_{i\text{,}j}+A_{i\text{,}j+1})\right)C_{i\text{,}j}^{n}\right]$$

This differential procedure is stable only if the following stability condition is met:

(13) $$\Delta t⩽\frac{\Delta x^{2}}{4D_{\max}}$$

### 2.4 Compartmental models

Three compartmental models the extended Tofts model, the parallel 3-compartment model, and the sequential 3-compartmental model are used to fit to the simulated concentration–time curves without taking either the blood signal of the simulation or the blood compartment of the models into account. For the total concentration, $C_{t}$, including the vascular compartment, the general solution of the extended Tofts model is obtained [3]:

(14) $$C_{t}(t)=K_{\mathrm{trans}}\int C_{p}(t^{\prime })e^{-(K_{\mathrm{trans}}/v_{e})(t-t^{\prime })}{\mathrm{dt}}^{\prime }+v_{p}C_{p}(t)\text{.}$$

Fitting of the Tofts model to the simulated contrast medium accumulation curves yields the model parameters corresponding to the simulation the transfer constant, $K_{\mathrm{trans}}$, and the relative interstitial volume, $v_{e}$.

Three-compartment models include the blood plasma compartment, $C_{p}$, and two interstitial compartments: the rapidly equilibrating compartment, $C_{\mathrm{ef}}$, with the corresponding relative interstitial volume $v_{\mathrm{ef}}$, and the slowly equilibrating compartment, $C_{\mathrm{es}}$, with the corresponding relative interstitial volume $v_{\mathrm{es}}$. Contrast medium accumulation in the two interstitial volumes occurs with two different transfer constants, $K_{\mathrm{transf}}$ and $K_{\mathrm{transs}}$. As with the Tofts model, exchange between the compartments occurs passively by diffusion. Taking the relative volumes of the three compartments into account, we obtain the total contrast medium concentration, $C_{t}$, in tissue using the following equation:

(15) $$C_{t}=v_{\mathrm{ef}}C_{\mathrm{ef}}+v_{\mathrm{es}}C_{\mathrm{es}}+v_{p}C_{p}\text{.}$$

In the following, we evaluate two three-compartment models: the parallel model [29] and the sequential model [30]. The *parallel* three-compartment model assumes that contrast medium exchange only takes place between blood and the two interstitial compartments but not between the two interstitial compartments:

(16) $$\frac{{\mathrm{dC}}_{\mathrm{ef}}}{\mathrm{dt}}=\frac{K_{\mathrm{transf}}}{v_{\mathrm{ef}}}\left(C_{p}-C_{\mathrm{ef}}\right)$$

(17) $$\frac{{\mathrm{dC}}_{\mathrm{es}}}{\mathrm{dt}}=\frac{K_{\mathrm{transs}}}{v_{\mathrm{es}}}\left(C_{p}-C_{\mathrm{es}}\right)$$

Unlike the parallel model, the *sequential* three-compartment model assumes that contrast medium first extravasates from the blood into the fast interstitial compartment and from the fast into the slow interstitial compartment. This model does not assume contrast medium exchange between blood and the slow interstitial compartment:

(18) $$\frac{{\mathrm{dC}}_{\mathrm{ef}}}{\mathrm{dt}}=\frac{K_{\mathrm{transf}}}{v_{\mathrm{ef}}}\left(C_{p}-C_{\mathrm{ef}}\right)$$

(19) $$\frac{{\mathrm{dC}}_{\mathrm{es}}}{\mathrm{dt}}=\frac{K_{\mathrm{transs}}}{v_{\mathrm{es}}}\left(C_{\mathrm{ef}}-C_{\mathrm{es}}\right)$$

These two three-compartment models separately determine the fast, $v_{\mathrm{ef}}$, and the slow, $v_{\mathrm{es}}$, interstitial volumes. The total interstitial volume is $v_{e}=v_{\mathrm{ef}}+v_{\mathrm{es}}$.

## 3 Simulation experiments

The computer simulations were programmed using the C++ programming language. First, the simulated area is defined as a square 2D spatial grid with side lengths *L*. The simulated area is discretized with grid widths Δx=Δy=1μm. The numerical solution of the diffusion equation within the simulated area using the difference scheme Eq. (12) allows calculation of the temporal and spatial indicator distribution in the area under investigation. The choice of the temporal steps, Δt, depends on the diffusion coefficient to make sure that the stability condition, (13), is met. Within the height of 1μm perpendicular to the two-dimensional simulation area, cylindrical symmetry is assumed. This assumption allows us to compare the results of the two-dimensional simulations with the results obtained with use of the three-dimensional compartmental models.

The indicator simulated is a commercially available low-molecular-weight extracellular contrast agent for clinical use in humans which is suitable for determining relative interstitial volume. Necrotic tumor tissue with an increased interstitial volume at a greater distance from feeding vessels is simulated using the two-dimensional tumor tissue model consisting of a square simulation area. The simulated vessel is located in the right upper corner of the simulated area. The distance of the vessel from the edges of the spatial grid in the corner is 20μm. The circular capillary has an inner radius of r=4μm with a vessel wall thickness of d=1μm, consistent with histometric results in tumor tissue [4].

The diffusion coefficient outside the capillary vessel was set to $D=485\;\mu {\text{m}}^{2}/\text{s}$, which is the diffusion constant identified for the contrast agent Gadoteric acid (Dotarem®, Guerbet, France) in [31]. The intravascular concentration of the contrast agent over time is given by a typical arterial input function (AIF), which was measured in pigs using Gadoteric acid [32]. The simulation duration, *T*, is 1000 s, which was chosen because it roughly corresponds to the longest acquisition time of a clinical MRI examination. The time step length of Δt of 0.0005s was chosen to satisfy the stability condition (Gl. 13).

**Fig. 2:**
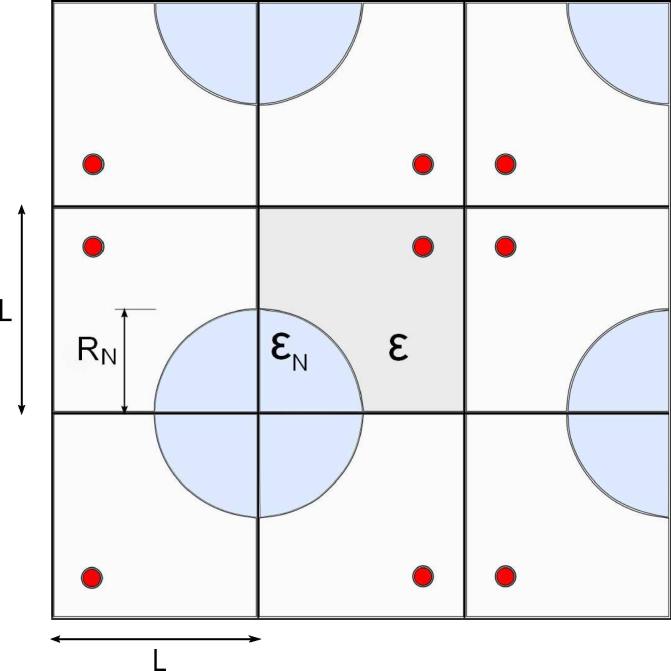
Using reflecting boundary conditions, an unbounded tumor tissue with circular blood capillaries drawn here in red is imitated. The simulated tissue is composed of squares with side length *L*. Porosity is defined in a position-dependent manner with $\varepsilon_{N}$ representing porosity within the necrotic core with radius $R_{N}$ and ε porosity in the remaining tissue outside the capillary.

Necrotic areas are simulated by enlarging the interstitial volume in a position-dependent manner, with $\varepsilon_{N}$ standing for porosity within the necrotic core with radius $R_{N}$ and ε for porosity in the remaining tissue outside the vessel. Simulations with reflecting boundary conditions were programmed to imitate an infinite inhomogeneous tissue with necrotic areas, see Fig. 2. The necrotic area with high $\varepsilon_{N}$ corresponds to the quarter circle with radius $R_{N}$ in each simulated square. As a result of the reflecting boundary condition, the necrotic areas are given as circular necrotic cores with radius $R_{N}$.

To determine the interstitial volume for a two-dimensional simulation, cylindrical symmetry is assumed along the longitudinal axis of the vessel, and the simulated area with height h=1μm is regarded as a three-dimensional space. The total simulated interstitial volume $v_{e}^{\mathrm{in}}$ can be calculated from the simulated interstitial volume, $\varepsilon_{N}$, in the necrotic core with radius $R_{N}$ and the interstitial volume ε outside the capillary as follows:

(20) $$\begin{array}{c} v_{e}^{\mathrm{in}}=\frac{\frac{1}{4}\pi R_{N}^{2}h}{L^{2}h}\varepsilon_{N}+\frac{L^{2}h-{\left(r+d\right)}^{2}\pi h-\frac{1}{4}\pi R_{N}^{2}h}{L^{2}h}\varepsilon \\ \;=\frac{\frac{1}{4}\pi R_{N}^{2}}{L^{2}}\varepsilon_{N}+\frac{L^{2}-{\left(r+d\right)}^{2}\pi -\frac{1}{4}\pi R_{N}^{2}}{L^{2}}\varepsilon \end{array}$$

The vessel radius is the outer radius of the capillary including capillary wall thickness (r+d) since the vessel wall is not part of the interstitial space. The first term of Eq. (20), the interstitial volume in the necrotic area, is $v_{\mathrm{es}}^{\mathrm{in}}$ and the second term, the interstitial volume in the remaining tissue, is $v_{\mathrm{ef}}^{\mathrm{in}}$. The two interstitial volumes refer to the entire volume of the simulated area.

In the simulation, interstitial volumes were varied both in the necrotic area and in the remaining tissue outside the blood capillary. For the relative interstitial volume outside the hypoxic area, values of ε= 5%,10%,15%,20%, and 25% were simulated. Within the necrotic area, porosities of $\varepsilon_{N}$ of 50% and 100% were simulated. Contrast medium diffusion was investigated for side lengths L=100μm and L=150μm in the simulated square to investigate long-distance diffusion in the necrotic areas. In addition, the radius of the necrotic area, $R_{N}$, is varied. For $L=100\;\mu \text{m}\text{,}\;R_{N}$ is varied between 10μm and 70μm. For L=150μm, it is varied between 10μm and 100μm at steps of 10μm.

Sodium fluorescein is a low-molecular-weight dye used as a fluorescent tracer. Its diffusion properties are comparable to those of the contrast medium Dotarem because its molecular weight (376 Da) is similar to that of gadoteric acid (559 Da). Additionally, both indicators are nonbinding extravascular extracellular tracers. Fu et al. [33] measured a permeability of approx. 0.3μm/s for sodium fluorescein. However, since vascular permeability is increased in tumor tissue [34], we set vascular permeability, *P*, to 1μm/s in the simulation. Thus, the mean interstitial indicator concentration at any time can be represented as a concentration-time curve, $C_{e}(t)$, as follows:

(21) $$C_{e}(t)=\frac{1}{N^{2}-\Pi {\left(r+d\right)}^{2}}\overset{N}{\underset{i\text{,}j=0}{\sum }}w_{i\text{,}j}\;\varepsilon (x_{i}\text{,}y_{j})C(x_{i}\text{,}y_{j}\text{,}t)$$

with

(22) $$\begin{array}{cc} & w_{i\text{,}j}=1\;\forall i\text{,}j\in v_{e}\\ & w_{i\text{,}j}=0\;\forall i\text{,}j\in v_{p}\text{.} \end{array}$$

The indicator concentration in the blood capillary is not included in the calculation in order to also ignore the blood plasma compartment when fitting the compartmental models. This means, for example, that for a 3-compartment model two compartments are fitted to the tissue curve calculated using Eq. (21).

The compartmental models are fitted to the concentration–time courses using a Fortran 77 program based on the simplex algorithm [35]. For side lengths L=100μm and L=150μm, a total of 70 and 100 simulations, respectively, were performed with variations in $\varepsilon \text{,}\;\varepsilon_{N}$, and $R_{N}$. Interstitial volumes of over 100% predicted by the models were excluded from further analysis since it is physically not possible for the interstitial volume to exceed this value. *N* is the number interstitial volumes below 100% predicted by each model.

## 4 Results

**Fig. 3:**
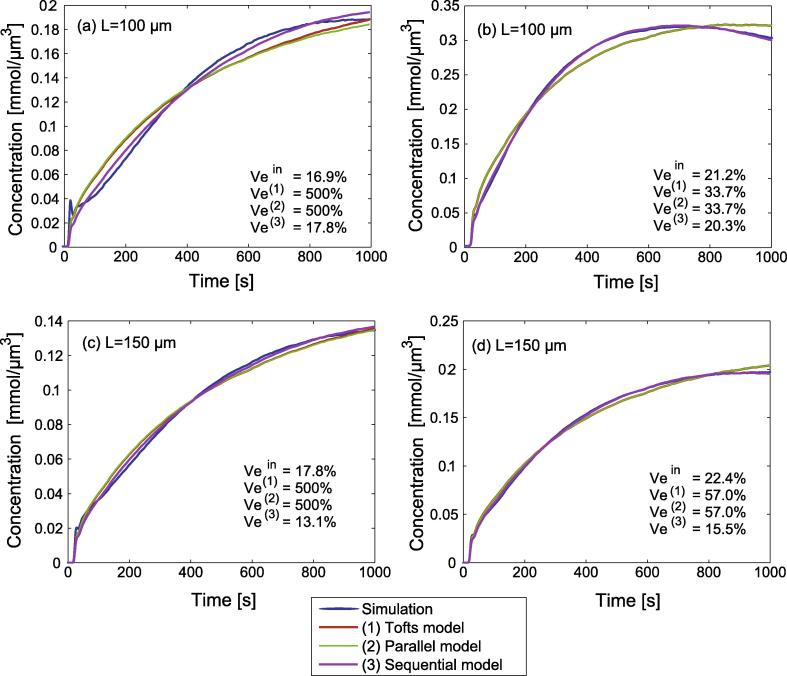
Exemplary fitting of the (1) Tofts model, the (2) parallel model, and the (3) sequential model to the concentration–time curves generated by the simulations for side lengths (a), (b) L=100μm and (c), (d) L=150μm. $v_{e}^{\mathrm{in}}$ is the predefined interstitial volume used in the simulation. $v_{e}^{\left(1\right)}\text{,}\;v_{e}^{\left(2\right)}\text{,}$ and $v_{e}^{\left(3\right)}$ are the interstitial volumes obtained with the respective compartmental models.

Fig. 3 illustrates the fitting of compartment models to different simulated concentration–time curves for simulated side lengths of L=100μm and L=150μm. $v_{e}^{\mathrm{in}}$ is the interstitial volume predefined in the simulation. $v_{e}^{\left(1\right)}\text{,}\;v_{e}^{\left(2\right)}\text{,}$ and $v_{e}^{\left(3\right)}$ stand for the total volumes obtained with the respective compartmental models.

In the examples shown, fitting with the sequential model yields the smallest deviation from the simulation compared with the Tofts model and the parallel 3-compartment model. The interstitial volumes determined by the Tofts model and the parallel model in Fig. 3(a) and (c) are above 100%, which is why these results were excluded from analysis.

Furthermore, the interstitial volumes, $v_{e}^{\mathrm{out}}$, predicted by the compartmental models used here are compared with the interstitial volumes, $v_{e}^{\mathrm{in}}$, used in the simulations. Since porosity in the simulated area is different for necrotic tissue and viable tissue outside the capillaries, we first look at the total interstitial volumes calculated from the simulations.

**Fig. 4:**
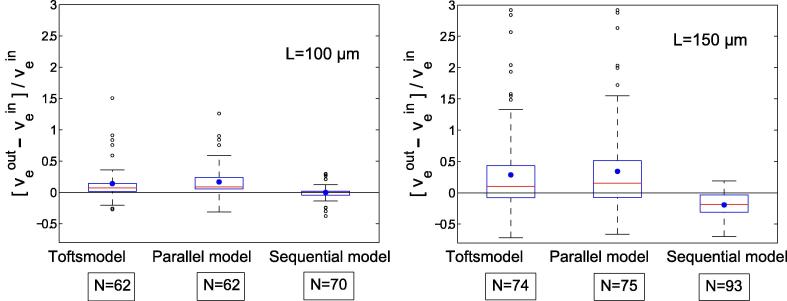
Box plots of the relative error in determining interstitial volume $v_{e}^{\mathrm{out}}$ using the Tofts model, the parallel model, and the sequential model from the given value, $v_{e}^{\mathrm{in}}$. The boxes represent the range in which half of the values determined are found. The box is limited by the upper and lower quartiles. The horizontal red line through the center of the box represents the median. The blue point is the mean. Values outside the box are illustrated by vertical lines. Outliers far away from the box are represented by open dots. *N* is the number of successful model fits with $v_{e}^{\mathrm{out}}<1.0$.

Fig. 4 presents box plots of the relative deviations of the interstitial volumes determined with the compartmental models for L=100μm and L=150μm from the simulations. Measures of distribution such as medians, upper and lower quartiles ($Q_{1}$ and $Q_{3}$), amd mean values (*M*) are summarized in Table 1. The number *N* in Fig. 4 and Table 1 is the number of simulations with $v_{e}^{\mathrm{out}}<1.0$ for each model.

Comparison of medians for the results in Fig. 4 for L=100μm shows that the total interstitial volume is overestimated by 6.9% with the Tofts model, by 8.6% with the parallel model, and by 0.2% with the sequential model. For the simulations with L=150μm, overestimation is 10% with the Tofts model and 15.5% with the parallel model. In contrast, the sequential model underestimates $v_{e}^{\mathrm{in}}$ by 18.8%. Looking at the mean relative errors (see Table 1), which are represented by blue dots in Fig. 4, shows that mean deviations are greater for the Tofts model and the parallel model compared with the sequential model.

**Fig. 5:**
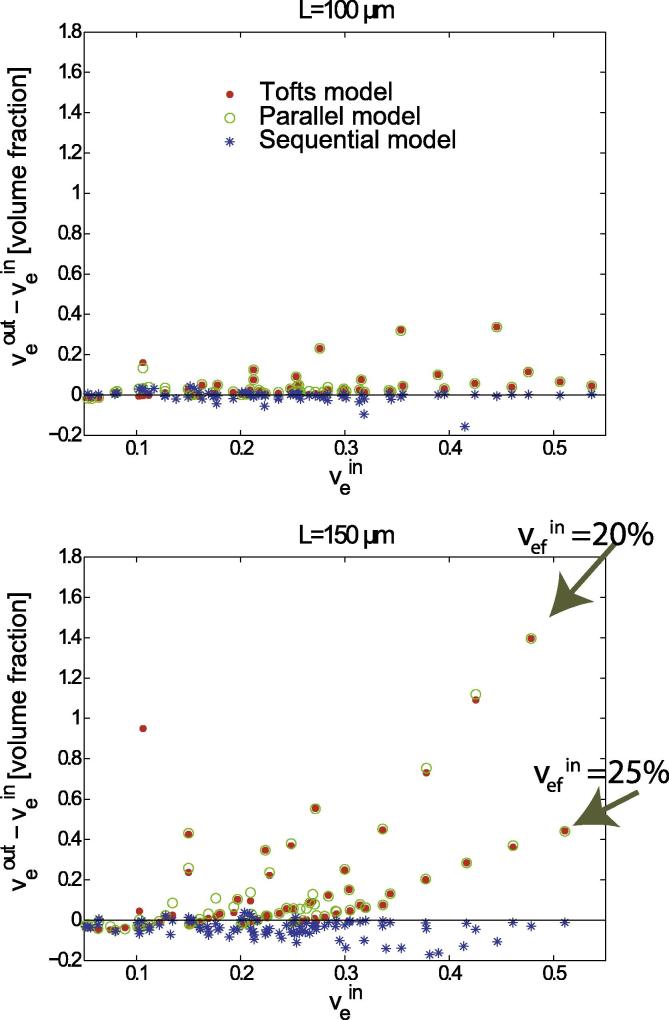
Absolute errors in determining relative interstitial volumes, $v_{e}^{\mathrm{out}}$, using the Tofts model, the parallel model, and the sequential compartmental model compared with the values used in the simulations, $v_{e}^{\mathrm{in}}$.

For both side lengths, *L*, of the simulated area, the deviations of the results obtained with the Tofts and parallel models are distributed over a wide range, whereas the deviations with the sequential model are distributed over a narrower range, see Fig. 4. The same was observed for the absolute error, $v_{e}^{\mathrm{out}}-v_{e}^{\mathrm{in}}$, displayed in Fig. 5 for simulations with L=100μm and L=150μm.

Overall, there is better agreement of $v_{e}^{\mathrm{out}}$ and $v_{e}^{\mathrm{in}}$ for the sequential compartmental model compared with the other two models investigated, see Fig. 4, Fig. 5. For L=100μm and L=150μm,70 and 100 simulations, respectively, were performed varying $\varepsilon \text{,}\varepsilon_{N}$, and $R_{N}$. A systematic increase in the overestimation of the fractional interstitial volume using the parallel and Tofts-model was found for $\varepsilon_{N}=100\%$, see arrows in Fig. 5b. The systematic increase was found increasing with diameters of the necrotic area $R_{N}=60$, 70,80,90, and 100μm. Fig. 5b demonstrates the effect for large rapid interstitial fractional volumes ($v_{\mathrm{ef}}^{\mathrm{in}}=$ 20%, and 25%). For smaller rapid interstitial fractional volumes ($v_{\mathrm{ef}}^{\mathrm{in}}=$ 5-15%) the absolute error is outside the range of the ordinate. The number of simulations *N* with physically meaningful results with $v_{e}^{\mathrm{out}}<1.0$ for L=100μm was 62 for the Tofts model and the parallel model versus 70 for the sequential model. For L=150μm, *N* was 74 for the Tofts model, 75 for the parallel model, and 93 for the sequential model.

For the comparison of the Tofts model and the parallel model at L=100μm, the *F*-test yielded a highly significant result for 23 of 70 curve fittings (p<0.01) in favor of the parallel model, and for 42 curves, the sum of erroneous squares was smaller for the Tofts model. Comparison of the fits between the Tofts model and the sequential model yielded a highly significant difference (p<0.01) for 65 concentration curves in favor of the sequential model, while, for 5 curves, the sum of erroneous square was smaller for the Tofts model.

For the analysis at L=150μm, the *F*-test yielded highly significant results (p<0.01) for 61 of 100 curve fittings when comparing the Tofts model and the parallel model and for 76 curve fittings when comparing the Tofts model and the sequential model. For 22 fits, the parallel model had more erroneous squares, and for 24 fits, the sequential model had more erroneous squares. Overall, the sequential model significantly better fits the concentration–time curves of simulated inhomogeneous tumor tissue compared with the Tofts model and the parallel model.

**Fig. 6:**
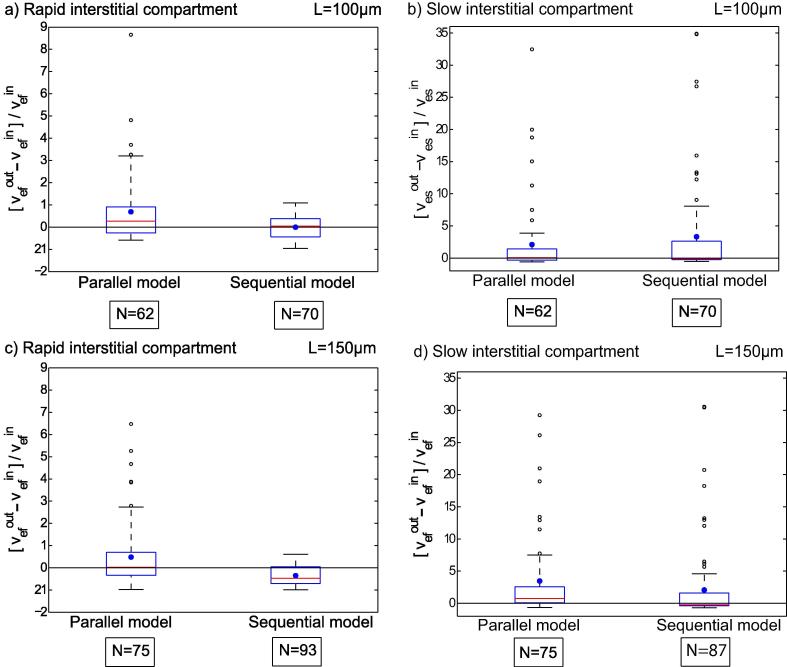
The rapid $v_{\mathrm{ef}}^{\mathrm{out}}$ and slow $v_{\mathrm{es}}^{\mathrm{out}}$ interstitial volumes predicted by the parallel and sequential compartmental models are compared with the interstitial volume in the remaining tissue outside necrotic areas, $v_{\mathrm{ef}}^{\mathrm{in}}$, and the interstitial volume in necrotic tissue, $v_{\mathrm{es}}^{\mathrm{in}}$, respectively. Figs. (a) and (b) present box plots of the relative errors for the rapidly and slowing equilibrating interstitial volumes for a simulated side length of L=100μm. For side length L=150μm, the corresponding box plots are presented in Figs. (c) and (d).

**Fig. 7:**
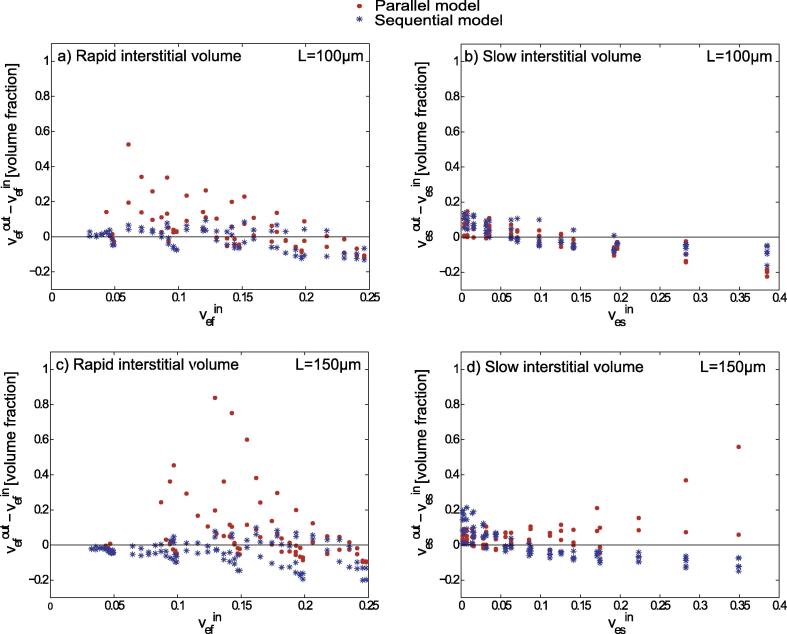
Graphic representation of the absolute errors in determination (a), (c) of the fast interstitial spaces, $v_{\mathrm{ef}}^{\mathrm{out}}$, relative to the interstitial volumes, $v_{\mathrm{ef}}^{\mathrm{in}}$, in simulated viable tissue near vessels and (b), (d) of the slow interstitial spaces, $v_{\mathrm{es}}^{\mathrm{out}}$, relative to the interstitial volumes, $v_{\mathrm{es}}^{\mathrm{in}}$, in simulated necrotic areas. Values were obtained for side lengths of the simulated area of L=100μm and L=150μm.

Furthermore, we compared the slowly equilibrating interstitial space, $v_{\mathrm{es}}^{\mathrm{out}}$, predicted by the parallel and sequential compartmental models with the simulated interstitial volume in the necrotic area, $v_{\mathrm{es}}^{\mathrm{in}}$. The rapidly equilibrating interstitial space, $v_{\mathrm{ef}}^{\mathrm{out}}$, was compared with the simulated interstitial volume in viable tissue, $v_{\mathrm{ef}}^{\mathrm{in}}$, see Fig. 6, Fig. 7. On average, the rapid interstitial volume was more severely overestimated by the parallel model compared with the sequential model. The sequential model overestimated the slow interstitial space for small necrotic interstitial volumes relative to the total volume, while it underestimated it as the necrotic interstitial volumes increased. Overall, deviations in determination of the slowly equilibrating interstitial space were smaller with the sequential model compared with the parallel model Table 2.

For L=100μm, the rapid interstitial volume is overestimated by 27.3% (median) using the parallel model and by 4.5% (median) using the sequential model. For L=150μm, however, the parallel model overestimates the rapid interstitial space by 2.2% (median), while the sequential model underestimates it by 47.2% (median). Overall, Fig. 6 shows large deviations, suggesting that the two 3-compartment models are limited and thus do not allow separate determination of interstitial volumes in necrotic areas and viable tissue in a reliable manner.

## 5 Discussion

We simulated contrast medium extravasation in heterogeneous tumor tissue to identify the compartmental model best suited to describe diffusion in inhomogeneous tissue. To account for the variation in interstitial volumes within a tumor, we determined interstitial volume in necrotic areas and viable tumor tissue around blood vessels. In our simulation experiments, we chose larger interstitial volumes to simulate necrotic areas and lower volumes for viable tumor areas. In addition, we varied the size of necrotic areas. The concentration–time curves derived in the experiments were assessed using three models: the 2-compartment Tofts model, and the parallel and sequential 3-compartment models. The compartment models were used to determine the total interstitial volume of the simulated tumor tissue. In generating the simulated concentration–time curves, blood plasma concentrations of contrast medium were ignored so we could neglect the blood plasma compartment in the compartment models. This allowed us to investigate contrast medium extravasation without having to account for other signal contributions such as intravascular signal. Since the extended Tofts model differs from the simple Tofts model only in the vascular contribution, the results of the current study are valid for both models. On the other hand fitting experimental tissue concentration time curves with signal contributions of both compartments would yield other fitting parameters for the same model.

Our results show that the sequential 3-compartmental model is more suitable than the other two models for assessing contrast medium dynamics in inhomogeneous tissues such as tumors, having the lowest relative and absolute error rates in determining interstitial volume. The *F*-test has shown that the sequential model significantly better fits the concentration–time curve in heterogeneous tissue than the other two models. Results indicating interstitial volumes over 100% were excluded from analysis. Overall, the number of results excluded for this reason was smallest for the sequential model. Moreover, the sequential 3-compartment model yields smaller absolute deviations in determining the rapidly and slowly equilibrating interstitial components compared with the parallel 3-compartment model. Thus, the sequential model has the greatest potential to separate the fast and slow interstitial volumes in dynamic magnetic resonance imaging. On the other hand the current study shows that the sequential 3-compartment model is only a rough approximation and has to be improved.

Some earlier investigations suggest that the pharmacokinetic behavior within tumors is more adequately described by 3-compartment models compared with 1-compartment or 2-compartment models [17], [29]. A 3-compartment model describes contrast medium extravasation by assuming a rapid and a slow phase. While the parallel 3-compartment model assumes contrast medium exchange between blood and each of the two interstitial compartments, the sequential model assumes contrast medium extravasation from the blood into the rapidly equilibrating compartment and from there into the slowly equilibrating compartment. Study [17] suggests that the sequential model better fits CM exchange in tumors. This is because it takes long-distance diffusion on the order of 200μm into account. Long-distance diffusion can take place into necrotic tumor areas and is accounted for by a transfer constant in 3-compartment models. Thus, the presence of slow extravasation in tumors may be attributable to the presence of micronecrotic and necrotic areas. Rapidly equilibrating interstitial space is present in all tumors and is assumed to characterize tissue around blood vessels.

The parameters assumed in 3-compartment models describe the tissue characteristics of tumors with the rapid parameters being assigned to viable tissue and the slow parameters being assigned to poorly supplied tissue such as necrotic areas. Separate assessment of slow and rapid extravasation components allows better characterization of tumors. Hence, the use of a 3-compartment model may possibly allow differentiation of necrotic and viable tumor components. However, the sequential 3-compartment model also yields results that significantly deviate from input values. Therefore, it would be desirable to have a model that explicitly takes diffusion into account. Such a model has been proposed by Pellerin et al. [18], which accounts for CM diffusion between voxels in small animal experiments. However, voxel dimensions are much larger in human applications, and contrast medium diffusion between voxels is negligible. Therefore, a model accounting for diffusion in humans remains to be developed.

## Figures and Tables

**Table 1:** Statistical data of the relative errors in determining interstitial volumes with the three compartmental models: Tofts model, parallel model, and sequential model. For the relative errors, the number of simulations *N*, the median, the upper quartile ($Q_{1}$), the lower quartile ($Q_{3}$), and the mean are given.

Parameter	Method	N	Median [%]	$Q_{1}$ [%]	$Q_{3}$ [%]	Mean [%]
L=100μm	Tofts model	62	6.9	1.6	14.0	14.0
	Parallel model	62	8.6	5.3	23.5	16.7
	Sequential model	70	0.2	−4.5	1.9	−0.2

L=150μm	Tofts model	74	10.0	−8.1	43.2	28.6
	Parallel model	75	15.5	−7.5	50.8	34.2
	Sequential model	93	−18.8	−31.1	−3.3	−19.3

**Table 2:** Statistical results of the relative errors in determining the rapid, $v_{\mathrm{ef}}$, and slow, $v_{\mathrm{es}}$, interstitial volumes using the parallel and sequential compartmental models. The table presents the number of simulations, *N*, median, and the mean (*M*).

*L*[μm]	Method	Parameter	N	Median [%]	*M* [%]
100	Parallel model	$v_{\mathrm{ef}}$	62	27.3	68.9
		$v_{\mathrm{es}}$	62	5.0	210.8
	Sequential model	$v_{\mathrm{ef}}$	70	4.5	−0.6
		$v_{\mathrm{es}}$	70	−11.1	332.4

150	Parallel model	$v_{\mathrm{ef}}$	75	2.2	48.6
		$v_{\mathrm{es}}$	75	74.5	346.3
	Sequential model	$v_{\mathrm{ef}}$	93	−47.2	−34.9
		$v_{\mathrm{es}}$	87	−22.2	206.7
